# Global decline in ocean memory over the 21st century

**DOI:** 10.1126/sciadv.abm3468

**Published:** 2022-05-06

**Authors:** Hui Shi, Fei-Fei Jin, Robert C. J. Wills, Michael G. Jacox, Dillon J. Amaya, Bryan A. Black, Ryan R. Rykaczewski, Steven J. Bograd, Marisol García-Reyes, William J. Sydeman

**Affiliations:** 1 Farallon Institute, Petaluma, CA 94952, USA.; 2 Department of Atmospheric Sciences, University of Hawaii, Honolulu, HI 96822, USA.; 3 Department of Atmospheric Sciences, University of Washington, Seattle, WA 98195, USA.; 4 Environmental Research Division, NOAA Southwest Fisheries Science Center, Monterey, CA 93940, USA.; 5 Physical Sciences Laboratory, NOAA Earth System Research Laboratories, Boulder, CO 80305, USA.; 6 Laboratory of Tree-Ring Research, University of Arizona, Tucson, AZ 85721, USA.; 7 Ecosystem Sciences Division, NOAA Pacific Islands Fisheries Science Center, Honolulu, HI 96818, USA.; 8 Department of Oceanography, University of Hawaii, Honolulu, HI 96822, USA.

## Abstract

Ocean memory, the persistence of ocean conditions, is a major source of predictability in the climate system beyond weather time scales. We show that ocean memory, as measured by the year-to-year persistence of sea surface temperature anomalies, is projected to steadily decline in the coming decades over much of the globe. This global decline in ocean memory is predominantly driven by shoaling of the upper-ocean mixed layer depth in response to global surface warming, while thermodynamic and dynamic feedbacks can contribute substantially regionally. As the mixed layer depth shoals, stochastic forcing becomes more effective in driving sea surface temperature anomalies, increasing high-frequency noise at the expense of persistent signals. Reduced ocean memory results in shorter lead times of skillful persistence-based predictions of sea surface thermal conditions, which may present previously unknown challenges for predicting climate extremes and managing marine biological resources under climate change.

## Teaser

Warming drives ocean memory loss leading to noisier, less predictable sea surface temperature variability.

## INTRODUCTION

More than two-thirds of Earth’s surface is covered by ocean, with a mostly thin (~50 m) layer of relatively warm, near-surface water on top of colder deep water. Despite the relative shallow depth of this surface mixed layer, the large specific heat of water in comparison to the overlying atmosphere results in sea surface temperatures (SSTs) varying much more slowly than the fast fluctuations of air temperature. The temporal persistence of anomalous ocean conditions—known as ocean memory and often measured by autocorrelation—has been noted as an important source of predictability in the climate system (*1*–*4*).

The depth of the upper-ocean mixed layer (MLD) is a key control on the persistence of SST anomalies on seasonal to interannual time scales. Deeper mixed layers have greater heat content, which confers thermal inertia, a source of memory that lengthens autocorrelation time scales of SST variability (*2*, *5*, *6*). The MLD is set by buoyancy contrasts between the surface water and the underlying deep water and is driven by mechanical stirring by the winds and buoyancy forcing at the surface (*7*, *8*). Processes at the air-sea interface and in the ocean act to dissipate or reinforce SST anomalies so as to modify their persistence, and they can be roughly categorized into two groups: (i) thermodynamic feedbacks (*9*–*12*) or recurring/persistent atmospheric circulation anomalies (*13*–*15*), which act through surface heat fluxes, and (ii) ocean dynamical processes, such as horizontal heat advection by ocean currents (*16*–*18*), entrainment or vertical mixing of waters at the base of the mixed layer, and reemergence due to seasonal variations of the MLD (*1*, *19*–*21*).

Observations and model projections for future scenarios show a reduction in the climatological MLD from continued greenhouse warming (*22*–*26*), mainly due to increasing upper-ocean stability (*27*). Here, using a comprehensive suite of Earth system models from the Coupled Model Intercomparison Project phase 6 (CMIP6) (*28*), we examine the hypothesis that this shoaling of the MLD may reduce upper-ocean memory in the coming decades, making annual mean SST less predictable. On the basis of a simple stochastic model of SST variability (*2*, *29*), we develop a mathematical expression that attributes the ocean memory decline primarily to changes in MLD, with secondary contributions from changes in air-sea feedbacks, mixing, and dynamical processes. Comparing the ocean memory decline with changes in SST variance, we further infer changes in the intensity of noise (i.e., random excitation of SST fluctuations) in the future climate system. Last, we discuss the implication of these findings for climate and ecosystem prediction.

## RESULTS

### Future change in year-to-year ocean memory

**Fig. 1.**
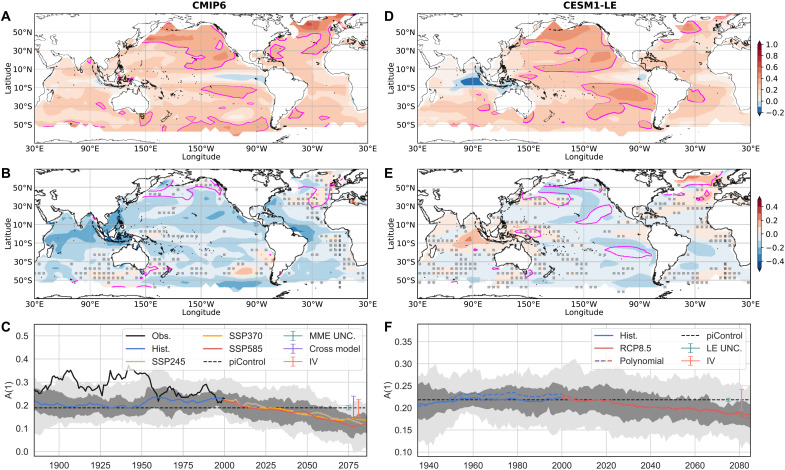
Declining year-to-year ocean memory through the 21st century. (**A**) Climatological 1-year autocorrelation, A(1), of annual SST anomalies at the end of the 19th century (1870–1899). The magenta lines bound regions of statistically significant A(1) (correlation = 0.3, degree of freedom = 28). (**B**) Change in A(1) from 1870–1899 to 2071–2100 under SSP5-8.5 scenario. Values are averaged over individual realizations from 20 different climate models from the CMIP6 multimodel ensemble (MME). The magenta lines bound regions of statistically significant A(1) in 2071–2100. Changes outside the gray dotted area are robust (Materials and Methods). White regions over ocean have seasonal or permanent sea-ice cover (Materials and Methods). (**C**) Global mean A(1) in 30-year rolling windows from observations and CMIP6 simulations from the historical and future (SSP) scenarios. Gray shadings show the range of values across models in percentiles: 25 to 75% (dark) and 5 to 95% (light). The dashed line is the A(1) averaged over the preindustrial control runs from the CMIP6 MME, with an error bar (cadet blue) showing the uncertainty in the MME (MME UNC.) (Materials and Methods). Error bars are also shown to quantify the cross-model spread (purple) and internal variability (salmon) (Materials and Methods). (**D** to **F**) Same as (A) to (C) but calculated with the 40-member Community Earth System Model Large Ensemble (CESM1-LE). (D) The climatological A(1) for 1920–1949 period and (E) change in A(1) between 1920–1949 and 2071–2100 in Representative Concentration Pathway (RCP) 8.5 scenario. In (F), the colored dashed line is the global mean A(1) with the fourth-order polynomial detrending, as used for the MME (Materials and Methods). Error bars show uncertainty in the ensemble mean (LE UNC.) and the spread due to internal variability (IV).

The 1-year autocorrelation of annual mean SST anomalies [hereafter referred to as A(1)] is used as a simple metric of the year-to-year ocean memory (Materials and Methods). The SST anomalies are defined as deviation from the long-term trends (Materials and Methods). The climatological A(1) is generally large and positive (up to 0.6 in CMIP6 models), except in the equatorial Eastern Pacific and parts of the Indo-Pacific warm pool. A(1) can even be negative in regions where the quasi-periodic climate modes such as the El Niño-Southern Oscillation (ENSO) and the Indian Ocean Dipole (IOD) dominate (Fig. 1A).

By the end of the 21st century, CMIP6 climate models project that A(1) will decrease throughout most of the world’s oceans under the Shared Socioeconomic Pathway SSP5-8.5 scenario, with some regions experiencing ocean memory reductions of up to 100% (Fig. 1B), as measured by A(1). Large areas of the North Pacific Ocean show a robust decrease in A(1), especially in the northeast, where the A(1) is reduced by about 50% on average. The western equatorial Atlantic Ocean, the North Atlantic Ocean off the U.S. east coast, the Caribbean Sea, and the mid-latitude South Atlantic Ocean also show reductions of A(1) of similar magnitude. An especially pronounced and broad-scale reduction in A(1) is projected to occur in the region spanning the Indian Ocean, South China Sea, and waters near the Maritime Continent (Fig. 1B).

These changes in A(1) are broadly reproduced within the Community Earth System Model Large Ensemble (CESM1-LE; Fig. 1E) (*30*), where 40 ensemble members with different realizations of internal variability allow for a better separation of the anthropogenically forced climate response and internal variability. In the CESM1-LE, a reduction in A(1) is found throughout the Pacific Ocean and South Atlantic Ocean, but the overall decrease is weaker than in the CMIP6 multimodel mean (Fig. 1E; cf. Fig. 1B). The CESM1-LE also shows isolated regions of increased A(1) in the North Atlantic and eastern Indian Ocean. The differences between CESM1-LE and the CMIP6 ensemble are particularly large in the Indian Ocean. These differences suggest that the amplitude and regional features of the memory change are model dependent. Despite some differences, both CMIP6 models and the CESM1-LE highlight common regions where, by the end of the 21st century, annual mean SST in 1 year will no longer be a significant predictor of annual mean SST in the following year. The size of the area with significant A(1) shrinks in the Pacific Ocean in particular. There is also a reduction in the area of significant A(1) in high latitudes of the North Atlantic (Fig. 1, A, B, D, and E).

A major decline in the global ocean memory is projected in three different future pathways in the CMIP6 simulations and in the CESM1-LE (Fig. 1, C and F). The trends of global mean A(1) (50°N to 50°S) from 2000 to 2100 range from −0.10 (*P* < 0.05, SSP3-7.0) to −0.13 (*P* < 0.05, SSP5-8.5) per century. Under continued greenhouse gas forcing, A(1) evolves similarly through the middle of the 21st century in all scenarios, after which A(1) begins to stabilize in SSP3-7.0. The global ocean memory decline qualitatively agrees with the shoaling trend of global mean MLD in model projections (fig. S1C), although there are some differences in the dominant regions of MLD shoaling and reduced A(1) (fig. S1B). It is worth noting that the observed ocean memory appears to be larger and more significant than those in the models (Fig. 1C and fig. S2), especially before around 1950. This may be partially due to the inadequacy in capturing relatively small-scale SST variance as a result of sampling in SST reconstruction.

While future anthropogenically forced changes in global ocean A(1) are apparent, there also exists a large degree of internal variability (Fig. 1, C and F, error bars and shading). However, as greenhouse gas concentrations continue to increase, the forced signal will become increasingly evident relative to this spread. Under the current trends, there would very likely be a historically unprecedented reduction in global ocean memory by the end of the 21st century.

### Processes contributing to ocean memory decline

We explore the mechanisms contributing to ocean memory decline using a simple stochastic model of SST variability (*2*), defining the damping rate (*r*) as an alternative way to represent A(1), where *r* = − ln *A*(1), and increases in damping rate correspond to decreases in ocean memory. This applies to all ocean regions where the SST variability is dominated by the red noise process. In some regions, other processes also affect the SST autocorrelation (e.g., in situ or remotely forced oscillatory variability), leading to very small or even negative A(1). In these cases, the damping rate is set to a constant corresponding to a threshold value of A(1) (Materials and Methods). We then use a mixed layer heat budget (Materials and Methods) to decompose damping rate changes (R) into contributions from three terms: (i) MLD changes (H), (ii) changes in the SST–surface–heat flux feedback (Q), and (iii) changes in ocean mixing and dynamics (M + D).

**Fig. 2.**
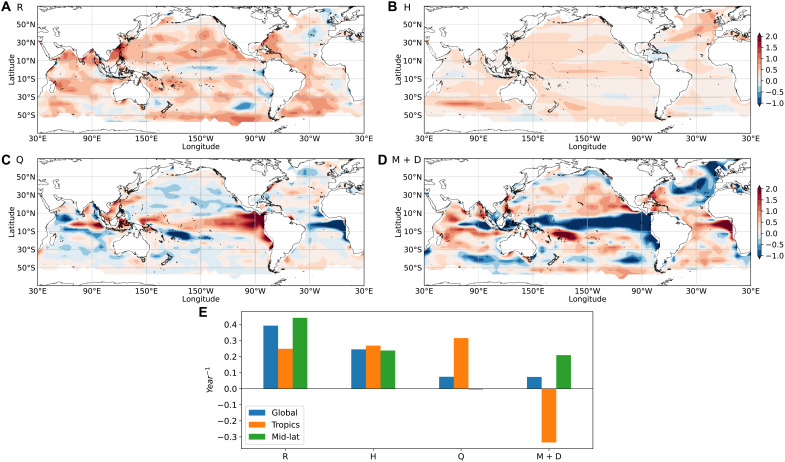
Causes of ocean memory decline. Changes from 1870–1899 to 2071–2100 in (**A**) damping rate (R, year^−1^); (**B**) MLD term (H); (**C**) SST–surface–heat flux feedback term (Q); (**D**) mixing and dynamic term (M + D); and (**E**) contribution of each term to R over the global oceans (50°N to 50°S), the tropics (10°N to 10°S), and mid-latitudes (10° to 50°N and S). R, H, Q, and M + D all represent changes. Heat fluxes, MLD, and SST data are from CMIP6 historical simulations and future projections under the SSP5-8.5 scenario.

Positive R is projected (i.e., reduced ocean memory) in most parts of the world’s oceans in CMIP6 future warming scenarios (Fig. 2A). In the tropical belt, R is less pronounced or negative compared with projected changes in the A(1) (Fig. 1B). This is because the A(1) changes in these regions involve both changes in the damping rate and the periodicity of tropical oscillatory modes. In these cases, the actual change in memory is more accurately represented by R (Fig. 2A). Admittedly, our crude determination of the damping rate in equatorial regions is subject to some errors (Materials and Methods), but these issues should not influence the extratropics, where A(1) is generally large. Averaged over the globe, ocean memory loss is quantified with an increase in the damping rate by 0.39 year^−1^ between 1870–1899 and 2071–2100. Stronger memory loss is found in the mid-latitudes (0.44 year^−1^), relative to the tropics (0.25 year^−1^; Fig. 2E).

The contribution of MLD changes (H) to R is predominantly positive (Fig. 2B) and best explains the increased damping rate over most of the global oceans (Fig. 2A). The substantial loss of upper-ocean thermodynamic memory is thus primarily driven by surface warming–induced shoaling of global MLDs, which reduces the effective heat capacity of the ocean surface layer. The reduced MLD and ocean memory are projected for both the winter and summer seasons (figs. S3 and S4), further confirming shoaling of MLD under year-round warming as the common mechanism, regardless of the seasonal variations of the MLD. Changes in SST–surface–heat flux feedback (Q) lead to large damping rate changes in the tropical oceans, most evidently in the equatorial eastern Pacific, Atlantic Niño, and IOD regions (Fig. 2C). In these regions, the ENSO, Atlantic Niño, and IOD are active, and the contributions from Q and M + D largely offset (Fig. 2, C and D). This cancellation between dynamics and thermodynamics is likely due to mean state changes that are known to generate similar cancellations in the ENSO growth rate simulated by climate models (*31*). Changes in ocean mixing and dynamics (M + D) contribute mostly in regions where active convection exists, e.g., in the Greenland-Iceland-Norwegian Seas, or in regions of strong upwelling, e.g., along the equator (Fig. 2D). The partial cancellation of H and M + D terms (Fig. 2, B and D) in the high-latitude North Atlantic likely indicates that a reduction in ocean heat transport convergence and vertical mixing contribute to the locally enhanced MLD shoaling in this region.

In the global mean, H is the main contributor to the increase in R (70%), followed by Q (21%), and M + D (9%). In the tropics, Q also contributes to the increase in R and is largely offset by the decrease in M + D. In the mid-latitudes, both H and M + D contribute to the R increase, and the contribution from Q is minimal. Therefore, on the global scale, future ocean memory decline is predominantly driven by future shoaling in mixed layer thermal inertia (i.e., the MLD), with the other feedbacks playing a relatively minor role. On the regional scale, however, both the SST–surface–heat flux feedback and ocean mixing and dynamics can contribute substantially to the memory decline together with the MLD changes.

### Consequences for SST variability

**Fig. 3.**
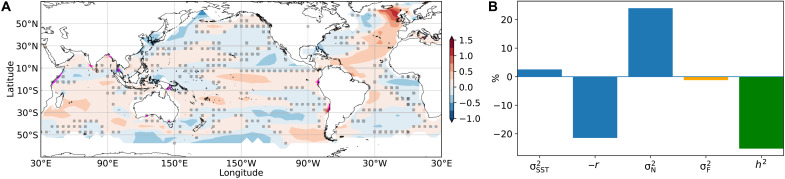
Changes in SST variance and signal-to-noise. (**A**) Fractional changes in SST variance from 2071–2100 to 1870–1899, with reference to the long-term mean variances of the entire period (1870–2100). Changes outside the gray dotted area are robust (Materials and Methods). (**B**) Global mean (50°N to 50°S) fractional changes in SST variance ($\sigma_{\text{SST}}^{2}$), damping rate (reversed, −*r*), noise variance ($\sigma_{N}^{2}$), variance of stochastic surface heat flux forcing ($\sigma_{F}^{2}$), and mix layer depth squared (*h*^2^). MLD and SST data are from CMIP6 historical simulations and future projections under the SSP5-8.5 scenario.

Year-to-year SST variations consist of two components: (i) slow variations due to ocean memory/persistence or remote forcing, which are reflected in a large autocorrelation and level of predictability, and (ii) noise, which are the random fluctuations associated with stochastic forcing and are largely unpredictable. To separate these components of SST variance, we use the Frankignoul and Hasselmann (*2*) model to compute an expression for the fractional change in annual SST variance ($\sigma_{\text{SST}}^{2}$) expressed as the difference between the fractional change of noise variance ($\sigma_{N}^{2}$) and that of the damping rate (*r*; Materials and Methods). While the persistent/predictable part of the overall SST variance decreases in the future (−21%), the increase in the noise/unpredictable part of the SST variance is larger (24%; Fig. 3B). As a result, the overall SST variance shows a slight increase (3%) in the future (Fig. 3, A and B).

Further decomposing $\sigma_{N}^{2}$ into a component that is driven by stochastic heat flux forcing (e.g., atmospheric turbulent heat fluxes) ($\sigma_{F}^{2}$, Materials and Methods) shows that the amplitude/variance of the stochastic forcing itself will decrease slightly (−1%) in the future (Fig. 3B). This suggests that the SSTs will become more sensitive to stochastic heat flux forcing (i.e., stochastic forcing becomes more effective at driving SST changes), such that $\sigma_{N}^{2}$ increases without an increase in the actual forcing.

Just as the shoaling of the MLD is the main reason for the decline in ocean memory, it is also the reason for the increase in noise variance (Materials and Methods). The reduced MLD (−25%; Fig. 3B) increases the effectiveness of stochastic forcing (i.e., heat fluxes at the ocean surface or mixed layer bottom) at generating SST anomalies, even when the amplitude of the forcing itself (i.e., the magnitude of heat flux anomalies) decreases. For example, although some studies have reported that the variance of the atmospheric winds become smaller under warming (*32*), they would more effectively lead to changes in SSTs because they are driving a shallower ocean mixed layer. The net result is a slight increase in year-to-year SST variability, and a reduced “signal-to-noise” ratio as the fraction of persistent/predictable SST variance is reduced in a warmer climate.

### Consequences for persistence-based predictions

**Fig. 4.**
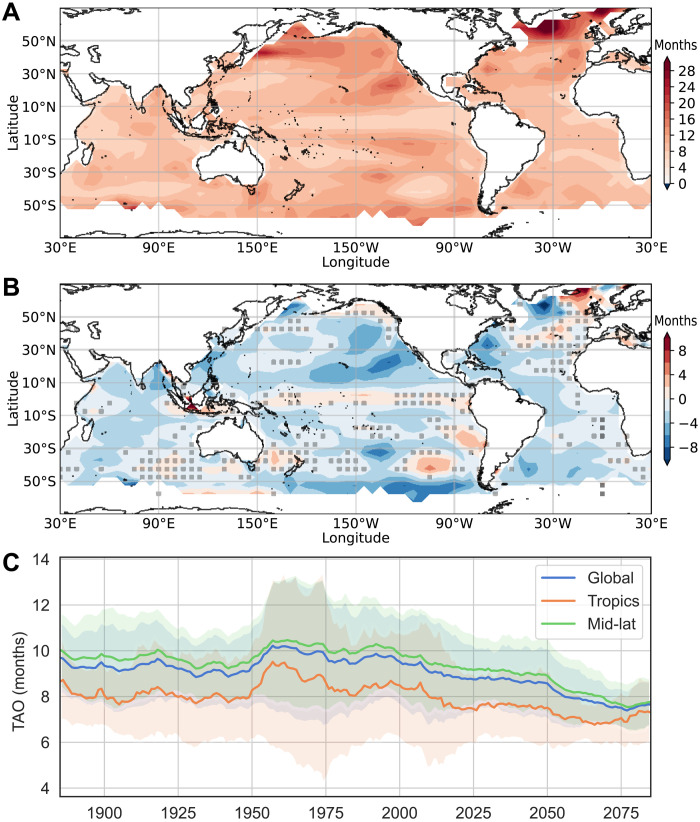
Projected declining ocean damping time scale. (**A**) Climatological damping time scale (TAO, months) at the end of the 19th century (1870–1899). (**B**) Projected change in damping time scale from 1870–1899 to 2071–2100 under SSP5-8.5 scenario. Changes outside the gray dotted area are robust (Materials and Methods). (**C**) Global and regional mean damping time scale in 30-year rolling windows under SSP5-8.5 scenarios. Shadings show 1 SD across models.

To estimate the impacts of memory decline on ocean predictions, we examined the changes in the damping time scale (Fig. 4), i.e., the inverse of the damping rate, which is an estimation of lead time for SST persistence predictions. For example, a damping time scale of 10 months would be equivalent to a lead time of 6.9 months for a persistence forecast correlation skill of 0.5. The climatological damping time scale ranges from 2 to 28 months (Fig. 4A). By the end of the 21st century following the SSP5-8.5 scenario, CMIP6 climate models project decreases in damping time scale over most of the world’s oceans (Fig. 4B). For the northeast Pacific Ocean and the western Atlantic Ocean, damping time scale is reduced by 6 to 8 months from originally 12 to 24 months. Globally, the damping time scale is reduced from an average of 9.7 to 7.7 months (Fig. 4C). This translates to a lead time change from 6.7 to 5.3 months for forecast skill of 0.5, meaning that the previously 2-quarter-lead forecast would drop to quarter-lead forecasts. The damping time scale reduction is slightly stronger for the mid-latitudes, which is 2.2 months from 10 to 7.9 months.

## DISCUSSION

This study described a projected steady decline of ocean memory over much of the global oceans throughout the 21st century. To explain the global decline in ocean memory and its consequences for SST variability, we provided an estimation of global changes in the SST damping rate, attribution of damping rate changes to the contributions from changes in MLD and thermal-dynamical feedbacks, and decomposition of the noise component of the SST variance. We found that globally the MLD shoaling contributes about two-thirds of the overall ocean memory decline. In the mid-latitudes, besides the shoaling of MLD, ocean mixing and dynamical processes also contribute substantially (about half) to the memory decline. In the tropics, all three processes substantially contribute to memory change, and the thermal and dynamical processes largely cancel each other. Estimates of the contributions of different processes are based on an idealized theoretical framework and use relatively limited data lengths with annual sampling, thus there remains some degree of uncertainty in these numbers, especially in regions where oscillatory climate modes or nonlocal dynamics (i.e., remotely forced SST changes) dominate. While there is a clear dominance of MLD changes over much of the globe, interesting questions about the other contributing processes remain to be explored, e.g., the reasons for opposite changes in SST–surface–heat flux feedbacks and SST variance in the Atlantic Niño region versus in the El Niño and IOD regions (Figs. 2C and 3A). Positive SST-low cloud feedbacks may decrease with warming in the major upwelling regions (*33*). However, this is only evident in the Peru Current region as an increase in Q (Fig. 2C). In the mid-latitude Pacific Ocean and South Atlantic Ocean, increases in M + D contribute notably to the increases in R (Fig. 2D), leading to slightly decreased SST variance in these regions (Fig. 3A). In-depth, regional-scale studies are needed to understand what specific dynamical processes are involved and how they will change under warming. This may also help to reconcile the reduction in year-to-year ocean memory reported in our study with the previously reported increase in week-to-week ocean memory (*34*).

The physical implications of ocean memory decline can be well understood through changes in the ocean damping time scale, which is directly associated with lead time for SST persistence predictions. Over many of the world’s large marine ecosystems (LMEs), SST anomalies are predictable at lead times of months to more than a year (*35*), enabling ecologically and societally relevant forecasts at seasonal-to-interannual time scales (*36*). Of particular interest is the accurate prediction of warm ocean extremes—known as marine heatwaves (MHWs)—which can markedly affect the distribution and productivity of marine species and the overall health of marine ecosystems (*37*–*43*). In most LMEs, the dominant source of SST predictability is persistence—or ocean memory—and differences in SST forecast skill between regions often reflect differences in persistence (*35*, *44*). Thus, the projected decline in ocean memory is likely to hinder ocean prediction efforts by reducing the lead times at which SST forecasts, including those for MHWs, are skillful. Future warming-induced MLD shoaling may also alter the statistics of temperature extremes, as the reduced thermal inertia of the mixed layer enables more rapid and pronounced temperature changes (*23*), which combined with reduced lead time for persistence-based predictions of ocean surface conditions will pose challenges for ecosystem management and marine hazard preparation.

In the terrestrial realm, seasonal-to-decadal predictions of temperature and rainfall usually draw substantial skill from SSTs (*45*–*48*). The persistence of SSTs is known to be a crucial factor for skillfully predicting monsoon variability (*49*–*52*) and terrestrial extremes, e.g., extreme summer precipitation (*53*), winter cold days (*54*, *55*), and heatwaves (*56*). Therefore, reduced SST persistence under warming likely renders previously identified predictability sources ineffective and requires searching for different sets of predictors. The previously unknown challenges in forecasting brought by ocean memory loss are crucial to address as we prepare for potentially more frequent and intense temperature and hydrological extremes in a warming world (*57*, *58*).

From an applied perspective, fisheries management relies on estimates of biological parameters to estimate stock size and set sustainable harvest rates. The demographic of fish, such as recruitment, is well known to be dependent on environmental conditions, including SST (*59*, *60*), although in most stock assessments they are assumed to be stable such that a “moving window” of estimates in the recent past is considered reflective of current environmental conditions (*61*). Less memory in ocean temperature may complicate that approach to management, potentially decreasing the accuracy of parameters used in stock assessments and management. In that case, there would be an increased need for alternative approaches in ecosystem-based fisheries management that aim to include near–real-time ocean monitoring and detailed understanding of environmental effects on fish population parameters.

The biological implications of changes in ocean memory are more uncertain, but consequential impacts on populations are likely. Some species with relatively constant reproductive effort, species with so-called K-selected life histories (*62*, *63*), are best suited for persistent environmental conditions and may flourish during periods of low variability. In contrast, the so-called r-selected species may “hedge their bets” and demonstrate multiple major reproductive efforts during years of rarely occurring optimal conditions (*64*). This fundamental dichotomy in species’ life history attributes may be useful for understanding and predicting which populations may be negatively or positively affected by future changes in ocean memory.

## MATERIALS AND METHODS

### Data

We use earth system model output from the CMIP6 (*28*) and the CESM1-LE (*30*). We use CMIP6 output of SST, MLD, surface latent heat flux, surface sensible heat flux, and surface radiative fluxes from the preindustrial control simulations, historical simulations, and various SSP (*65*) scenarios, including SSP2-4.5, SSP3-7.0, and SSP5-8.5. We selected a total of 20 CMIP6 models (table S1) that have all targeted variables across all experiments. We also use SST data from 40 ensemble members of the CESM1-LE historical and RCP (Representative Concentration Pathway) (*66*) 8.5 simulations.

The observational data we used include the monthly SST data from Met Office Hadley Center’s sea ice and sea-surface temperature dataset (HadISST) from 1870 to 2019, with 1° resolution (*67*). Historical SST observations (1982–2019) from the National Oceanic and Atmospheric Admistration Optimum Interpolation SST, version 2 (OISSTv2) (*68*, *69*) were also used to identify seasonal and permanent sea ice cover. Regions where OISSTv2 ice concentrations were greater than zero for more than 15 days in any month were masked out in all analyses to excluding memory changes due to changes in sea ice properties. All data were interpolated onto a 5° grid for analysis.

### Year-to-year ocean memory

The lag-1 autocorrelations A(1) of annual (January to December) SST anomalies were used to represent year-to-year ocean memory. The A(1) metric is applicable for most parts of the world oceans. In the equatorial regions, where interannual oscillatory modes tend to dominate the SST variability, the simple persistence metric A(1) does not capture predictability associated with the periodicity of these modes. Therefore, we made modifications when calculating the damping rate in these regions (next section).

To remove the long-term forced response in the CMIP6 data, we removed a fourth-order polynomial trend from SST data in both model simulations (1870 to 2100) and observational data (1870 to 2014) as in Hawkins and Sutton (*70*) [see also (*71*)]. We then calculate the climatological A(1) at the end of the 19th century (1870–1899) and the epoch difference of A(1) between 1870–1899 and 2071–2100. Area-weighted A(1) in a 30-year rolling window is calculated over the global oceans to demonstrate the time variations of observed and simulated A(1). We look at changes in the multimodel-ensemble mean (MME) and use piControl simulations, i.e., long simulations with greenhouse gas forcing fixed at preindustrial levels, to quantify uncertainties due to sampling of internal variability. We calculate the A(1) in a total of eight nonoverlapping 30-year periods from the piControl simulations and calculate the multimodel mean uncertainty as the SD of the multimodel means across these eight preindustrial control periods. When the MME change between the future and historical exceeds 1 SD of the multimodel mean, the change is considered robust. We also calculate the SD across the 20 models (in each of the eight periods) to quantify the cross-model spread. The SD across the eight periods (in each of the 20 models) represents the spread in multimodel-mean A(1) due to internal variability.

For the CESM-LE, the forced response is estimated by the ensemble-mean SST and is removed from the individual ensemble members. We also calculate the forced response using the fourth-order polynomial used for the CMIP6 data and find consistent results (Fig. 1F). There is no cross-model spread for the LE. The internal variability is represented by the SD across the 40 members, and the uncertainty in the ensemble mean is the square root of the total variance due to internal variability divided by 40 (the number of ensemble members).

### Attribution of ocean memory change

According to the stochastic climate model (*2*, *29*), SST anomalies evolve according to a red noise process

$$\frac{dT^{\prime }}{\mathrm{dt}}=-rT^{\prime }+N,$$ (1)

Here, *T*^′^ is the SST anomaly, *r* is the damping rate, and *N* represents white noise process (in units of °C s^−1^). The damping time scale (*r*^−1^) quantifies the ocean memory. The damping rate can be related to A(1), where *r* takes the unit of year^−1^

r=−ln A(1) .

In regions where A(1) is negative or very close to zero [A(1) < 0.05], we estimate *r* as follows

$$r=\{\begin{array}{c} -\text{ln}\;(0.05),\text{for}\;10{}^{\circ}-50{}^{\circ}N\;\text{and}\;S\\ -\text{ln}\;(\text{max}(|A(1)|,0.05)),\text{for}\;10{}^{\circ}S-10{}^{\circ}N \end{array}$$

In the mid-latitudes, we assume the red noise process dominates, and we use a threshold A(1) to avoid obtaining unrealistically large *r*. For the tropics, where the oscillatory process is important, we estimate the envelope of the SST autocorrelations by using the larger absolute value of A(1) and the threshold A(1) to calculate *r*. We chose the threshold of A(1) = 0.05 because it is the critical correlation at the 5% significance level for degree of freedom = 30 − 2, and it satisfies the sampling frequency requirement [A(1) > > 0.01; next section]. Using threshold A(1) may cause underestimation of *r* in the mid-latitudes, especially for the end of the 21st century, when 31% of data points in the mid-latitudes have A(1) smaller than 0.05. However, the percentage only decreased slightly to 28% when reducing the threshold A(1) to 0.03, indicating that reducing the threshold A(1) will not substantially enlarge regions included in mid-latitude calculations. To fully separate the red noise and the oscillatory processes, more sophisticated methods are required, which is beyond the scope of this paper. Overall, our approach is a simple way to account for the oscillatory nature of tropical coupled modes, and it provides a reasonable estimation of the ocean memory in the tropical region; it also yields a smoother long-term mean damping rate ($\bar{r}$) globally by avoiding adding noise from regions with low A(1).

To quantify the different processes that contribute to changes in the damping time scale and A(1), we examine the heat budget of the ocean mixed layer

$$\frac{dT^{\prime }}{\mathrm{dt}}=\frac{Q^{\prime }-{\left(W_{E}(T-T_{b})\right)}^{\prime }}{C_{P}\rho h}+D^{\prime }+Rsd.$$

Here, *Q*^′^ is the surface heat flux anomaly, (*W*_E_(*T* − *T*_b_))^′^ is the turbulent entrainment flux anomalies (or mixing), where *W*_E_ is the entrainment velocity, *T* and *T*_b_ are the temperature in and at the bottom of the mixed layer, respectively. *D*^′^ + *Rsd* are ocean dynamics and subgrid-scale residuals, and *h* is the climatological MLD. Constants ρ and *C*_P_ are the density and heat capacity of seawater, respectively. We parameterize the surface heat fluxes *Q*^′^ and the ocean dynamic terms of this equation in terms of temperature-dependent feedbacks α_Q_ and α_M + D_ and noise *N*_Q_ and *N*_M + D_ whereby we obtain

$$\frac{Q^{\prime }}{C_{P}\rho h}=-\frac{\alpha_{Q}}{h}T^{\prime }+N_{Q}$$ (2)

and

$$-\frac{{\left(W_{E}(T-T_{b})\right)}^{\prime }}{C_{P}\rho h}+D^{\prime }+Rsd=-\frac{\alpha_{M+D}}{h}T^{\prime }+N_{M+D},$$ (3)

Here, we assume that ocean dynamics and subscale effects can be represented by a combined mixing flux anomaly. Summing Eqs. 2 and 3 and comparing with Eq. 1, *r* can be expressed in terms of a temperature-dependent feedback parameter α

$$r=\alpha /h.,\;\text{where}\;\alpha =\alpha_{Q}+\alpha_{M+D}$$

A significant change in A(1) implies a significant change in *r*. Thus, we express *r* in two parts after linearization, i.e., the long-term mean ($\bar{r}$) and the change/trend (∆*r*)

$$r=\bar{r}+∆r.$$

The change in *r* can be further decomposed into components due to changes in MLD (∆*h*), changes in the SST–surface–heat flux feedback (∆α_Q_), and changes in ocean dynamics and mixing (∆α_M + D_)

$$∆r=-\bar{r}\frac{∆h}{\bar{h}}+\frac{∆\alpha_{Q}}{\bar{h}}+\frac{∆\alpha_{M+D}}{\bar{h}}$$

{R} {H} {Q} {M+D}

The first term (H) is diagnosed by calculating the relative change ($\frac{\text{∆}h}{\bar{h}}$) in MLD between 1870–1899 and 2071–2100 periods in reference to the long-term mean MLD. The long-term mean damping rate ($\bar{r}$) was obtained by averaging the calculated damping rate in 30-year windows across the 1870–2100 period. The second term (Q) is diagnosed by calculating the changes in surface heat flux feedbacks (∆α_Q_) between 1870–1899 and 2071–2100 periods through linear regression based on Eq. 2 and weighting the change by the long-term mean MLD. The third term (M + D) is diagnosed as a residual. All diagnostics are done grid by grid over the world oceans.

Here, our estimation of the SST–surface–heat flux feedback is based on the assumption that surface heat fluxes depend on the local SST, an assumption that is embedded in the Frankignoul-Hasselmann model. Surface heat flux changes due to nonlocal cloud and circulation changes (e.g., teleconnections from ENSO) will not be captured by this term (they will be captured by the M + D term instead). While these dynamic effects may alter the picture on the regional scale, the estimations of contributions to ocean memory loss on the global or hemispheric scale will be robust.

### Change in variance of noise and stochastic forcing

SST varies on a continuum of time scales. Changes in ocean memory influence SST variability differently on different time scales. From the stochastic climate model (*2*, *29*), we can take a Fourier transform of Eq. 1 to obtain the temperature variance as a function of frequency

$$\sigma_{T}^{2}(f)=\frac{\sigma_{N}^{2}}{r^{2}}\frac{1}{1+4\pi^{2}r^{-2}f^{2}}.$$

Integrating over frequencies between 0 and the Nyquist frequency *f_N_* leads to a relationship between the variance of annual mean SST anomalies ($\sigma_{T}^{2}$) and the variance of the noise ($\sigma_{N}^{2}$)

$$\sigma_{T}^{2}=\frac{\sigma_{N}^{2}}{2\pi r}{\text{tan}}^{-1}(2\pi r^{-1}f_{N}).$$

In the high-frequency sampling limit (*r* ≪ π^2^*f_N_*)

$$\sigma_{T}^{2}=\frac{\sigma_{N}^{2}}{4r}\;.$$

For the annual anomalies studied here (i.e., *f_N_* = 0.5 year^−1^), this is approximately true for *r* ≪ 4.9 [note that *r* = 4.9 corresponds to A(1) = 0.007]. We can then infer the fractional changes in $\sigma_{N}^{2}$ as

$$\frac{∆\sigma_{N}^{2}}{\bar{\sigma_{N}^{2}}}=\frac{∆\sigma_{T}^{2}}{\bar{\sigma_{T}^{2}}}+\frac{∆r}{\bar{r}}$$

where $\bar{\sigma_{N}^{2}}$, $\bar{\sigma_{T}^{2}}$, and $\bar{r}$ are the climatological means, and $\text{∆}\sigma_{N}^{2}$, $\text{∆}\sigma_{T}^{2}$, and ∆*r* are changes.

The noise *N* (in units of °C year^−1^) can be understood as resulting from stochastic forcing (heat flux from the surface and the bottom of the mixed layer) *F* of an ocean mixed layer with effective heat capacity *C* = ρ*C*_P_*h*, where *N* and *F* have the relationship $N=\frac{F}{C}$. Therefore, the variance of the stochastic heat flux forcing $\sigma_{F}^{2}$can be expressed as

$$\sigma_{F}^{2}=C^{2}\sigma_{N}^{2}.$$

Fractional changes in $\sigma_{F}^{2}$ can therefore be expressed as

$$\frac{∆\sigma_{F}^{2}}{\bar{\sigma_{F}^{2}}}=\frac{∆\sigma_{N}^{2}}{\bar{\sigma_{N}^{2}}}+\frac{{\Delta h}^{2}}{\bar{h^{2}}}$$

where $\bar{\sigma_{N}^{2}}$, $\bar{\sigma_{F}^{2}}$, and $\bar{h^{2}}$ are the climatological means, and $\text{∆}\sigma_{N}^{2}$, $\text{∆}\sigma_{F}^{2}$, and ∆*h*^2^ are changes. Therefore, while we find a large increase in $\sigma_{N}^{2}$, this results from a decrease in the MLD rather than an increase in the stochastic heat flux forcing $\sigma_{F}^{2}$.

### Statistical analysis

The statistical significance of A(1) is calculated with the two-tailed *t* test for Pearson correlation with the degree of freedom that equals to the sample size minus two (30 − 2 years in the study). The statistical significance of the global ocean memory trends (*P* values in the text) is calculated with a Monte Carlo method, which takes into consideration the relatively high autocorrelation of the global averaged A(1) time series. We calculated the trends in 5000 time series generated with the same level of autocorrelation as those of the global mean A(1) time series and obtained the significance level of the trends through ranking.

## Supplementary Materials

### This PDF file includes:

Figs. S1 to S4 Table S1 References

## References

[1] J. Namias, R. M. Born, Temporal coherence in North Pacific sea-surface temperature patterns. J. Geophys. Res. 75, 5952–5955 (1970).

[2] C. Frankignoul, K. Hasselmann, Stochastic climate models, Part II application to sea-surface temperature anomalies and thermocline variability. Tellus. 29, 289–305 (1977).

[3] C. Deser, M. A. Alexander, M. S. Timlin, Understanding the persistence of sea surface temperature anomalies in midlatitudes. J. Climate 16, 57–72 (2003).

[4] A. Srivastava, T. DelSole, Decadal predictability without ocean dynamics. Proc. Natl. Acad. Sci. U.S.A. 114, 2177–2182 (2017). 28193900 10.1073/pnas.1614085114 PMC5338527

[5] J. Namias, X. Yuan, D. R. Cayan, Persistence of North Pacific sea surface temperature and atmospheric flow patterns. J. Climate 1, 682–703 (1988).

[6] G. de Coëtlogon, C. Frankignoul, The persistence of winter sea surface temperature in the North Atlantic. J. Climate 16, 1364–1377 (2003).

[7] K. E. Brainerd, M. C. Gregg, Surface mixed and mixing layer depths. Deep. Res. Part I 42, 1521–1543 (1995).

[8] H. W. Wijesekera, M. C. Gregg, Surface layer response to weak winds, westerly bursts, and rain squalls in the western Pacific warm pool. J. Geophys. Res. C Ocean. 101, 977–997 (1996).

[9] C. Frankignoul, Sea surface temperature anomalies, planetary waves, and air-sea feedback in the middle latitudes. Rev. Geophys. 23, 357 (1985).

[10] C. Frankignoul, E. Kestenare, The surface heat flux feedback, Part I: Estimates from observations in the Atlantic and the North Pacific. Clim. Dynam. 19, 633–647 (2002).

[11] S. Park, C. Deser, M. A. Alexander, Estimation of the surface heat flux response to sea surface temperature anomalies over the global oceans. J. Climate 18, 4582–4599 (2005).

[12] J. J. Barsugli, D. S. Battisti, The basic effects of atmosphere-ocean thermal coupling on midlatitude variability. J. Atmos. Sci. 55, 477–493 (1998).

[13] X. Zhao, J. Li, Winter-to-Winter recurrence of sea surface temperature anomalies in the Northern hemisphere. J. Climate 23, 3835–3854 (2010).

[14] X. Zhao, J. Li, Winter-to-winter recurrence and non-winter-to-winter recurrence of SST anomalies in the central North Pacific. J. Geophys. Res. Ocean. 117, C12023 (2012).

[15] P. Byju, D. Dommenget, M. A. Alexander, Widespread reemergence of sea surface temperature anomalies in the global oceans, Including tropical regions forced by reemerging winds. Geophys. Res. Lett. 45, 7683–7691 (2018).

[16] O. Leeuwenburgh, D. Stammer, The effect of ocean currents on sea surface temperature anomalies. J. Phys. Oceanogr. 31, 2340–2358 (2001).

[17] N. Schneider, B. D. Cornuelle, The forcing of the Pacific decadal oscillation. J. Climate 18, 4355–4373 (2005).

[18] C. H. O’Reilly, L. Zanna, The signature of oceanic processes in decadal extratropical SST anomalies. Geophys. Res. Lett. 45, 7719–7730 (2018).

[19] M. A. Alexander, C. Deser, A mechanism for the recurrence of wintertime midlatitude SST anomalies. J. Phys. Oceanogr. 25, 122–137 (1995).

[20] M. A. Alexander, C. Deser, M. S. Timlin, The reemergence of SST anomalies in the North Pacific Ocean. J. Clim. 12, 2419–2433 (1999).

[21] K. Hanawa, S. Sugimoto, “Reemergence” areas of winter sea surface temperature anomalies in the world’s oceans. Geophys. Res. Lett. 31, 1–4 (2004).

[22] M. A. Alexander, J. D. Scott, K. D. Friedland, K. E. Mills, J. A. Nye, A. J. Pershing, A. C. Thomas, Projected sea surface temperatures over the 21^st^ century: Changes in the mean, variability and extremes for large marine ecosystem regions of Northern Oceans. Elementa. 6, 9 (2018).

[23] D. J. Amaya, M. A. Alexander, A. Capotondi, C. Deser, K. B. Karnauskas, A. J. Miller, N. J. Mantua, Are long-term changes in mixed layer depth influencing North Pacific Marine heatwaves? Bull. Am. Meteorol. Soc. 102, S59–S66 (2021).

[24] A. Capotondi, M. A. Alexander, N. A. Bond, E. N. Curchitser, J. D. Scott, Enhanced upper ocean stratification with climate change in the CMIP3 models. J. Geophys. Res. Ocean. 117, C04031 (2012).

[25] H. J. Freeland, Evidence of change in the winter mixed layer in the Northeast Pacific Ocean: A problem revisited. Atmos. - Ocean. 51, 126–133 (2013).

[26] F. Liu, J. Lu, Y. Luo, Y. Huang, F. Song, On the oceanic origin for the enhanced seasonal cycle of SST in the midlatitudes under global warming. J. Climate 33, 8401–8413 (2020).

[27] G. Li, L. Cheng, J. Zhu, K. E. Trenberth, M. E. Mann, J. P. Abraham, Increasing ocean stratification over the past half-century. Nat. Clim. Chang. 10, 1116–1123 (2020).

[28] V. Eyring, S. Bony, G. A. Meehl, C. A. Senior, B. Stevens, R. J. Stouffer, K. E. Taylor, Overview of the coupled model intercomparison project phase 6 (CMIP6) experimental design and organization. Geosci. Model Dev. 9, 1937–1958 (2016).

[29] K. Hasselmann, Stochastic climate models Part I. Theory. Tellus. 28, 473–485 (1976).

[30] J. E. Kay, C. Deser, A. Phillips, A. Mai, C. Hannay, G. Strand, J. M. Arblaster, S. C. Bates, G. Danabasoglu, J. Edwards, M. Holland, P. Kushner, J. F. Lamarque, D. Lawrence, K. Lindsay, A. Middleton, E. Munoz, R. Neale, K. Oleson, L. Polvani, M. Vertenstein, The community earth system model (CESM) large ensemble project: A community resource for studying climate change in the presence of internal climate variability. Bull. Am. Meteorol. Soc. 96, 1333–1349 (2015).

[31] T. Bayr, C. Wengel, M. Latif, D. Dommenget, J. Lübbecke, W. Park, Error compensation of ENSO atmospheric feedbacks in climate models and its influence on simulated ENSO dynamics. Climate Dynam. 53, 155–172 (2019).

[32] J. Ma, S. P. Xie, Y. Kosaka, Mechanisms for tropical tropospheric circulation change in response to global warming. J. Climate 25, 2979–2994 (2012).

[33] T. Andrews, J. M. Gregory, M. J. Webb, The dependence of radiative forcing and feedback on evolving patterns of surface temperature change in climate models. J. Clim. 28, 1630–1648 (2015).

[34] J. Li, D. W. J. Thompson, Widespread changes in surface temperature persistence under climate change. Nature 599, 425–430 (2021). 34789900 10.1038/s41586-021-03943-z

[35] C. A. Stock, K. Pegion, G. A. Vecchi, M. A. Alexander, D. Tommasi, N. A. Bond, P. S. Fratantoni, R. G. Gudgel, T. Kristiansen, T. D. O’Brien, Y. Xue, X. Yang, Seasonal sea surface temperature anomaly prediction for coastal ecosystems. Prog. Oceanogr. 137, 219–236 (2015).

[36] M. G. Jacox, M. A. Alexander, S. Siedlecki, K. Chen, Y. O. Kwon, S. Brodie, I. Ortiz, D. Tommasi, M. J. Widlansky, D. Barrie, A. Capotondi, W. Cheng, E. Di Lorenzo, C. Edwards, J. Fiechter, P. Fratantoni, E. L. Hazen, A. J. Hermann, A. Kumar, A. J. Miller, D. Pirhalla, M. P. Buil, S. Ray, S. C. Sheridan, A. Subramanian, P. Thompson, L. Thorne, H. Annamalai, K. Aydin, S. J. Bograd, R. B. Griffis, K. Kearney, H. Kim, A. Mariotti, M. Merrifield, R. Rykaczewski, Seasonal-to-interannual prediction of North American coastal marine ecosystems: Forecast methods, mechanisms of predictability, and priority developments. Prog. Oceanogr. 183, 102307 (2020).

[37] N. A. Bond, M. F. Cronin, H. Freeland, N. Mantua, Causes and impacts of the 2014 warm anomaly in the NE Pacific. Geophys. Res. Lett. 42, 3414–3420 (2015).

[38] L. M. Cavole, A. M. Demko, R. E. Diner, A. Giddings, I. Koester, C. M. L. S. Pagniello, M. L. Paulsen, A. Ramirez-Valdez, S. M. Schwenck, N. K. Yen, M. E. Zill, P. J. S. Franks, Biological impacts of the 2013–2015 warm-water anomaly in the northeast Pacific: Winners, losers, and the future. Oceanography 29, 273–285 (2016).

[39] M. G. Jacox, M. A. Alexander, N. J. Mantua, J. D. Scott, G. Hervieux, R. S. Webb, F. E. Werner, Forcing of multiyear extreme ocean temperatures that impacted California current living marine resources in 2016. Bull. Am. Meteorol. Soc. 99, S27–S33 (2018).

[40] J. F. Piatt, J. K. Parrish, H. M. Renner, S. K. Schoen, T. T. Jones, M. L. Arimitsu, K. J. Kuletz, B. Bodenstein, M. García-Reyes, R. S. Duerr, R. M. Corcoran, R. S. A. Kaler, G. J. McChesney, R. T. Golightly, H. A. Coletti, R. M. Suryan, H. K. Burgess, J. Lindsey, K. Lindquist, P. M. Warzybok, J. Jahncke, J. Roletto, W. J. Sydeman, Extreme mortality and reproductive failure of common murres resulting from the northeast Pacific marine heatwave of 2014-2016. PLOS ONE 15, e0226087 (2020). 31940310 10.1371/journal.pone.0226087 PMC6961838

[41] D. J. Amaya, A. J. Miller, S. P. Xie, Y. Kosaka, Physical drivers of the summer 2019 North Pacific marine heatwave. Nat. Commun. 11, 1903 (2020). 32313028 10.1038/s41467-020-15820-w PMC7171163

[42] D. A. Smale, T. Wernberg, E. C. J. Oliver, M. Thomsen, B. P. Harvey, S. C. Straub, M. T. Burrows, L. V. Alexander, J. A. Benthuysen, M. G. Donat, M. Feng, A. J. Hobday, N. J. Holbrook, S. E. Perkins-Kirkpatrick, H. A. Scannell, A. Sen Gupta, B. L. Payne, P. J. Moore, Marine heatwaves threaten global biodiversity and the provision of ecosystem services. Nat. Clim. Chang. 9, 306–312 (2019).

[43] M. G. Jacox, M. A. Alexander, S. J. Bograd, J. D. Scott, Thermal displacement by marine heatwaves. Nature 584, 82–86 (2020). 32760046 10.1038/s41586-020-2534-z

[44] G. Hervieux, M. A. Alexander, C. A. Stock, M. G. Jacox, K. Pegion, E. Becker, F. Castruccio, D. Tommasi, More reliable coastal SST forecasts from the North American multimodel ensemble. Climate Dynam. 53, 7153–7168 (2019).

[45] M. Hoerling, A. Kumar, The perfect ocean for drought. Science (80-. ). 229, 691–694 (2003). 10.1126/science.1079053 12560548

[46] F. J. Doblas-Reyes, J. García-Serrano, F. Lienert, A. P. Biescas, L. R. L. Rodrigues, Seasonal climate predictability and forecasting: Status and prospects. Wiley Interdiscip. Rev. Clim. Chang. 4, 245–268 (2013).

[47] S. D. Schubert, R. E. Stewart, H. Wang, M. Barlow, E. H. Berbery, W. Cai, M. P. Hoerling, K. K. Kanikicharla, R. D. Koster, B. Lyon, A. Mariotti, C. R. Mechoso, O. V. Müller, B. Rodriguez-Fonseca, R. Seager, S. I. Senevirante, L. Zhang, T. Zhou, Global meteorological drought: A synthesis of current understanding with a focus on sst drivers of precipitation deficits. J. Climate 29, 3989–4019 (2016).

[48] D. M. Smith, A. A. Scaife, B. P. Kirtman, What is the current state of scientific knowledge with regard to seasonal and decadal forecasting? Environ. Res. Lett. 7, 015602 (2012).

[49] B. Wang, J. Y. Lee, B. Xiang, Asian summer monsoon rainfall predictability: A predictable mode analysis. Climate Dynam. 44, 61–74 (2014).

[50] B. Wang, B. Xiang, J. Li, P. J. Webster, M. N. Rajeevan, J. Liu, K. J. Ha, Rethinking Indian monsoon rainfall prediction in the context of recent global warming. Nat. Commun. 6, 7154 (2015). 25981180 10.1038/ncomms8154 PMC4479044

[51] S. Y. Yim, B. Wang, W. Xing, Peak-summer East Asian rainfall predictability and prediction part II: Extratropical East Asia. Climate Dynam. 47, 15–30 (2016).

[52] W. Xing, B. Wang, S. Y. Yim, K. J. Ha, Predictable patterns of the May-June rainfall anomaly over East Asia. J. Geophys. Res. 122, 2203–2217 (2017).

[53] J. Li, B. Wang, Predictability of summer extreme precipitation days over eastern China. Climate Dynam. 51, 1–12 (2018).

[54] X. Luo, B. Wang, How predictable is the winter extremely cold days over temperate East Asia? Climate Dynam. 48, 2557–2568 (2017).

[55] X. Luo, B. Wang, Predictability and prediction of the total number of winter extremely cold days over China. Climate Dynam. 50, 1769–1784 (2018).

[56] M. Gao, B. Wang, J. Yang, W. Dong, Are peak summer sultry heat wave days over the Yangtze-Huaihe River basin predictable? J. Climate 31, 2185–2196 (2018).

[57] I. M. Held, B. J. Soden, Robust responses of the hydrological cycle to global warming. J. Climate 19, 5686–5699 (2006).

[58] D. L. Swain, B. Langenbrunner, J. D. Neelin, A. Hall, Increasing precipitation volatility in twenty-first-century California. Nat. Clim. Chang. 8, 427–433 (2018).

[59] N. C. Stenseth, A. Mysterud, G. Ottersen, J. W. Hurrell, K.-S. Chan, M. Lima, Ecological effects of climate fluctuations. Science (80-. ). 297, 1292–1296 (2002). 10.1126/science.1071281 12193777

[60] P. van der Sleen, P. A. Zuidema, J. Morrongiello, J. L. J. Ong, R. R. Rykaczewski, W. J. Sydeman, E. Di Lorenzo, B. A. Black, Interannual temperature variability is a principal driver of low-frequency fluctuations in marine fish populations. Commun. Biol. 5, 28 (2022). 35017642 10.1038/s42003-021-02960-y PMC8752724

[61] A. E. Punt, T. A’mar, N. A. Bond, D. S. Butterworth, C. L. de Moor, J. A. A. De Oliveira, M. A. Haltuch, A. B. Hollowed, C. Szuwalski, Fisheries management under climate and environmental uncertainty: Control rules and performance simulation. ICES J. Mar. Sci. 71, 2208–2220 (2014).

[62] E. R. Pianka, On r-and K-selection. Am. Nat. 104, 592–597 (1970).

[63] D. Reznick, M. J. Bryant, F. Bashey, r- and K-selection revisited: The role of population regulation in life-history evolution. Ecology 83, 1509–1520 (2002).

[64] S. Hočevar, J. A. Hutchings, A. Kuparinen, Multiple-batch spawning as a bet-hedging strategy in highly stochastic environments: An exploratory analysis of Atlantic cod. Evol. Appl. 14, 1980–1992 (2021). 34429743 10.1111/eva.13251 PMC8372085

[65] B. C. O’Neill, C. Tebaldi, D. P. Van Vuuren, V. Eyring, P. Friedlingstein, G. Hurtt, R. Knutti, E. Kriegler, J. F. Lamarque, J. Lowe, G. A. Meehl, R. Moss, K. Riahi, B. M. Sanderson, The Scenario Model Intercomparison Project (ScenarioMIP) for CMIP6. Geosci. Model Dev. 9, 3461–3482 (2016).

[66] J. F. Lamarque, P. P. Kyle, M. Meinshausen, K. Riahi, S. J. Smith, D. P. van Vuuren, A. J. Conley, F. Vitt, Global and regional evolution of short-lived radiatively-active gases and aerosols in the representative concentration pathways. Clim. Change 109, 191–212 (2011).

[67] N. A. Rayner, Global analyses of sea surface temperature, sea ice, and night marine air temperature since the late nineteenth century. J. Geophys. Res. 108, 4407 (2003).

[68] R. W. Reynolds, T. M. Smith, C. Liu, D. B. Chelton, K. S. Casey, M. G. Schlax, Daily high-resolution-blended analyses for sea surface temperature. J. Climate 20, 5473–5496 (2007).

[69] V. Banzon, T. M. Smith, T. Mike Chin, C. Liu, W. Hankins, A long-term record of blended satellite and in situ sea-surface temperature for climate monitoring, modeling and environmental studies. Earth Syst. Sci. Data 8, 165–176 (2016).

[70] E. Hawkins, R. Sutton, The potential to narrow uncertainty in regional climate predictions. Bull. Am. Meteorol. Soc. 90, 1095–1108 (2009).

[71] F. Lehner, C. Deser, N. Maher, J. Marotzke, E. M. Fischer, L. Brunner, R. Knutti, E. Hawkins, Partitioning climate projection uncertainty with multiple large ensembles and CMIP5/6. Earth Syst. Dynam. 11, 491–508 (2020).

[72] A. Storto, S. Masina, C-GLORSv5: An improved multipurpose global ocean eddy-permitting physical reanalysis. Earth Syst. Sci. Data 8, 679–696 (2016).

