# Supplementary material for: Global decline in ocean memory over the 21st century
Source: Sci Adv. 2022 May 6;8(18):eabm3468. doi: 10.1126/sciadv.abm3468 (PMC11633086; doi:10.1126/sciadv.abm3468)
Supplement: Supplementary file 1 — Figs. S1 to S4 Table S1 References [file sciadv.abm3468_sm.pdf]

Science Advances
AAAS

Supplementary Materials for

Global decline in ocean memory over the 21st century

Hui Shi*, Fei-Fei Jin*, Robert C. J. Wills, Michael G. Jacox, Dillon J. Amaya, Bryan A. Black, Ryan R. Rykaczewski, Steven J. Bograd, Marisol García-Reyes, William J. Sydeman

*Corresponding author. Email: daisyhuishi@faralloninstitute.org (H.S.); jff@hawaii.edu (F.-F.J.)

Published 6 May 2022, *Sci. Adv.* **8**, eabm3468 (2022)
DOI: 10.1126/sciadv.abm3468

This PDF file includes:

Figs. S1 to S4
Table S1
References

Figure S1. Shoaling mixed layer depth (MLD) through the 21st century. a. Climatological MLD for the end of the 19th century (1870-1899). b. Change in MLD from 1870-1899 to 2071-2100 under SSP5-8.5 scenario. Values are averaged over individual realizations from 20 different climate models from the CMIP6 multi-model ensemble (MME). Changes outside the gray dotted area are robust (methods). c. Global-mean MLD in 30-yr rolling windows from CMIP6 simulations for the historical and future (SSP) scenarios. Gray shadings show the range of values across models in percentiles: 25%–75% (dark) and 5%–95% (light). The dashed line is the MLD averaged over the preindustrial control runs from the CMIP6 MME, with an error bar (cadet blue) showing the uncertainty in the MME (methods). Error bars are also shown to quantify the cross-model spread (purple) and internal variability (salmon) (methods). The model MLD is bias corrected by subtracting the climatological monthly MLD (1980-2014) from each model at each grid point to ensure the same historical climatology across the models. The reanalysis MLD data is the Global Ocean Physics Reanalysis C-GLORS v5 (*72*) obtained from http://c-glors.cmcc.it/index/index.html.

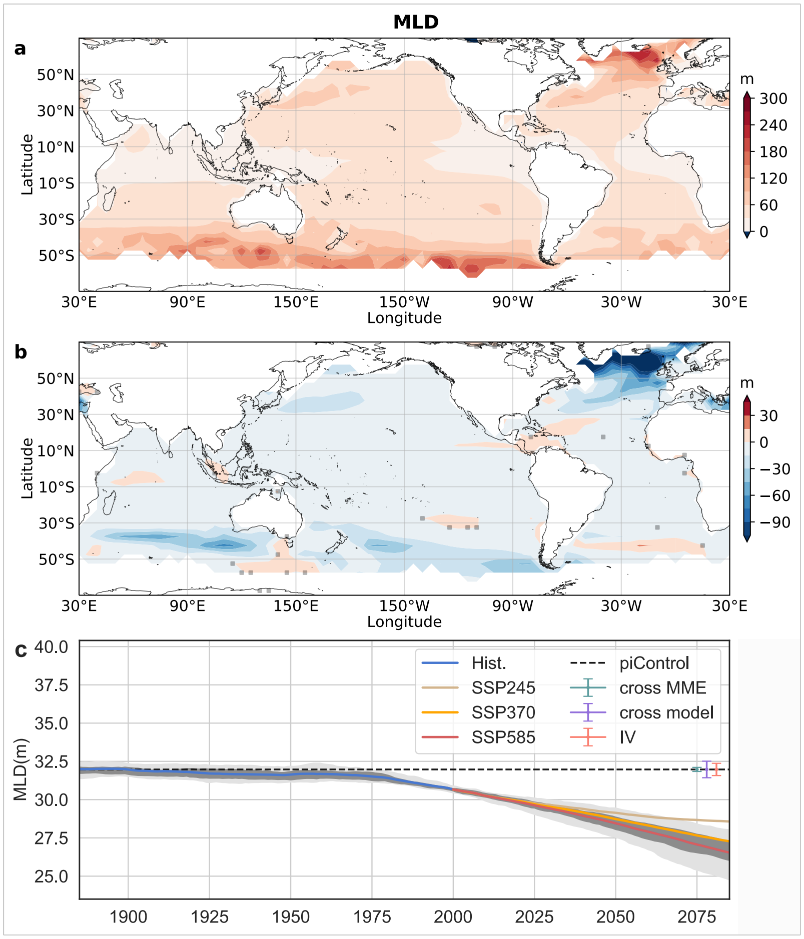

Figure S2. Observed (a) and CMIP6 model simulated (b) climatological one-year autocorrelation, A(1), of annual SST anomalies during 1961-2014. The magenta lines bound regions of statistically significant A(1) (correlation=0.23, DOF=48). White regions over ocean have seasonal or permanent sea-ice cover (methods). Observational data is the HadISST data obtained from the UK Met Office (https://www.metoffice.gov.uk/hadobs/hadisst/).

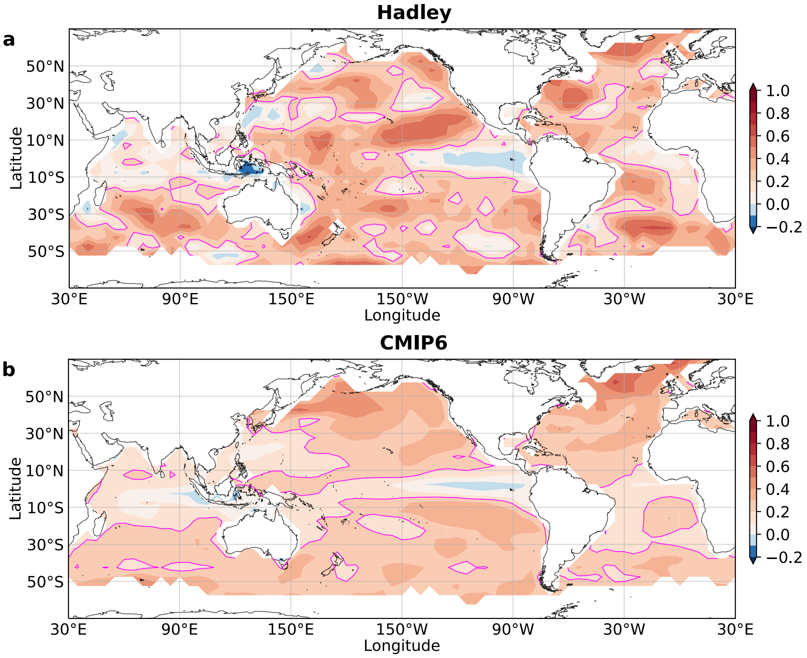

Figure S3. Changes in ocean memory for boreal winter (Dec-Jan-Feb, DJF, left column) and summer (Jun-Jul-Aug, JJA, right column). a.d. Climatological one-year autocorrelation, A(1), of SST anomalies at the end of the 19th century (1870-1899). b.e. Same as a.b but for future projection (2071-2100) under SSP5-8.5 scenario. The magenta lines mark statistically significant A(1) (correlation=0.3, DOF=28). c.f. Change in A(1) from 1870-1899 to 2071-2100 under SSP5-8.5 scenario. The magenta lines mark statistically significant A(1) in 2071-2100. Changes outside the gray dotted area are robust (methods). All values are from CMIP6 MME.

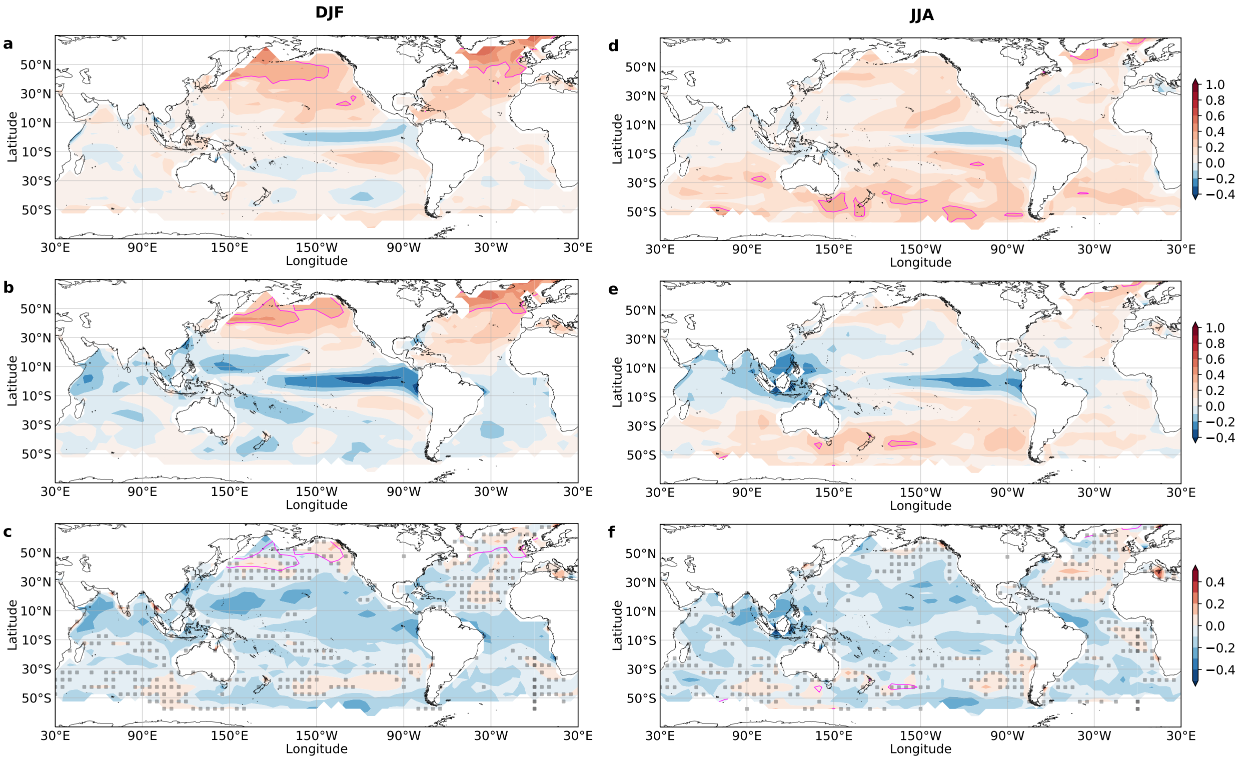

Figure S4. Same as Fig. S1a, b but for MLD changes in boreal winter (Dec-Jan-Feb, DJF, left column) and summer (Jun-Jul-Aug, JJA, right column) from 1870-1899 to 2071-2100 under SSP5-8.5 scenario in the CMIP6 multi-model ensemble.

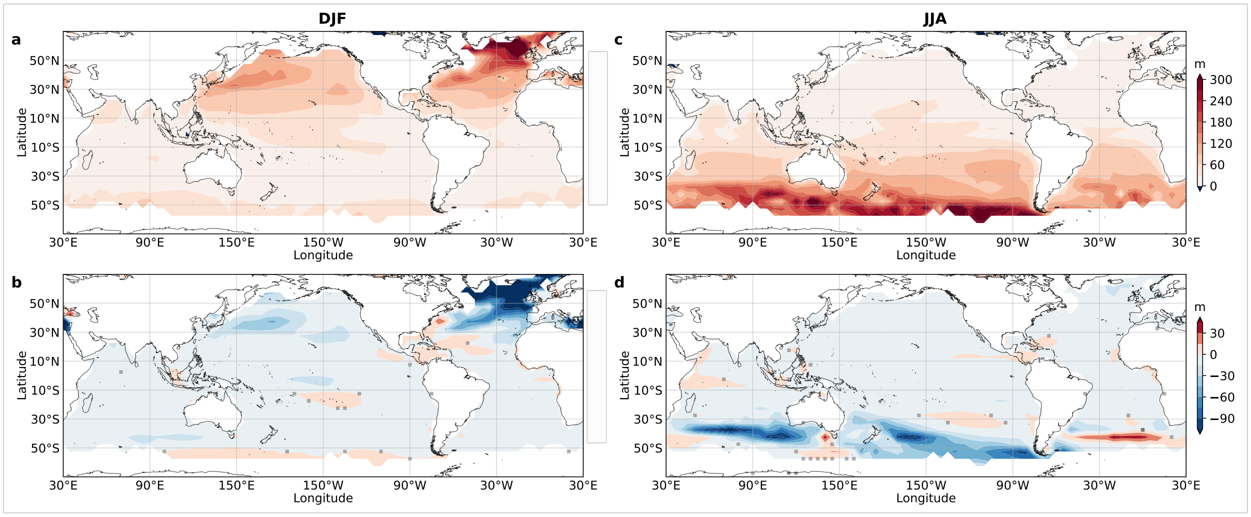

Table S1. CMIP6 models used for ensemble analysis

No.	Model name	No.	Model name
1	ACCESS-CM2	11	EC-Earth3
2	ACCESS-ESM1-5	12	EC-Earth3-Veg
3	AWI-CM-1-1-MR	13	GFDL-ESM4
4	BCC-CSM2-MR	14	IPSL-CM6A-LR
5	CAMS-CSM1-0	15	MPI-ESM1-2-HR
6	CanESM5	16	MPI-ESM1-2-LR
7	CESM2	17	MRI-ESM2-0
8	CESM2-WACCM	18	NorESM2-LM
9	CNRM-CM6-1	19	NorESM2-MM
10	CNRM-ESM2-1	20	UKESM1-0-LL

REFERENCES AND NOTES

1. J. Namias, R. M. Born, Temporal coherence in North Pacific sea-surface temperature patterns. *J. Geophys. Res.* **75**, 5952–5955 (1970).

2. C. Frankignoul, K. Hasselmann, Stochastic climate models, Part II application to sea-surface temperature anomalies and thermocline variability. *Tellus.* **29**, 289–305 (1977).

3. C. Deser, M. A. Alexander, M. S. Timlin, Understanding the persistence of sea surface temperature anomalies in midlatitudes. *J. Climate* **16**, 57–72 (2003).

4. A. Srivastava, T. DelSole, Decadal predictability without ocean dynamics. *Proc. Natl. Acad. Sci. U.S.A.* **114**, 2177–2182 (2017).

5. J. Namias, X. Yuan, D. R. Cayan, Persistence of North Pacific sea surface temperature and atmospheric flow patterns. *J. Climate* **1**, 682–703 (1988).

6. G. de Coëtlogon, C. Frankignoul, The persistence of winter sea surface temperature in the North Atlantic. *J. Climate* **16**, 1364–1377 (2003).

7. K. E. Brainerd, M. C. Gregg, Surface mixed and mixing layer depths. *Deep. Res. Part I* **42**, 1521–1543 (1995).

8. H. W. Wijesekera, M. C. Gregg, Surface layer response to weak winds, westerly bursts, and rain squalls in the western Pacific warm pool. *J. Geophys. Res. C Ocean.* **101**, 977–997 (1996).

9. C. Frankignoul, Sea surface temperature anomalies, planetary waves, and air-sea feedback in the middle latitudes. *Rev. Geophys.* **23**, 357 (1985).

10. C. Frankignoul, E. Kestenare, The surface heat flux feedback. Part I: Estimates from observations in the Atlantic and the North Pacific. *Clim. Dynam.* **19**, 633–647 (2002).

11. S. Park, C. Deser, M. A. Alexander, Estimation of the surface heat flux response to sea surface temperature anomalies over the global oceans. *J. Climate* **18**, 4582–4599 (2005).

12. J. J. Barsugli, D. S. Battisti, The basic effects of atmosphere-ocean thermal coupling on midlatitude variability. *J. Atmos. Sci.* **55**, 477–493 (1998).

13. X. Zhao, J. Li, Winter-to-Winter recurrence of sea surface temperature anomalies in the Northern hemisphere. *J. Climate* **23**, 3835–3854 (2010).

14. X. Zhao, J. Li, Winter-to-winter recurrence and non-winter-to-winter recurrence of SST anomalies in the central North Pacific. *J. Geophys. Res. Ocean.* **117**, C12023 (2012).

15. P. Byju, D. Dommenget, M. A. Alexander, Widespread reemergence of sea surface temperature anomalies in the global oceans, Including tropical regions forced by reemerging winds. *Geophys. Res. Lett.* **45**, 7683–7691 (2018).

16. O. Leeuwenburgh, D. Stammer, The effect of ocean currents on sea surface temperature anomalies. *J. Phys. Oceanogr.* **31**, 2340–2358 (2001).

17. N. Schneider, B. D. Cornuelle, The forcing of the Pacific decadal oscillation. *J. Climate* **18**, 4355–4373 (2005).

18. C. H. O'Reilly, L. Zanna, The signature of oceanic processes in decadal extratropical SST anomalies. *Geophys. Res. Lett.* **45**, 7719–7730 (2018).

19. M. A. Alexander, C. Deser, A mechanism for the recurrence of wintertime midlatitude SST anomalies. *J. Phys. Oceanogr.* **25**, 122–137 (1995).

20. M. A. Alexander, C. Deser, M. S. Timlin, The reemergence of SST anomalies in the North Pacific Ocean. *J. Clim.* **12**, 2419–2433 (1999).

21. K. Hanawa, S. Sugimoto, "Reemergence" areas of winter sea surface temperature anomalies in the world's oceans. *Geophys. Res. Lett.* **31**, 1–4 (2004).

22. M. A. Alexander, J. D. Scott, K. D. Friedland, K. E. Mills, J. A. Nye, A. J. Pershing, A. C. Thomas, Projected sea surface temperatures over the 21st century: Changes in the mean,

variability and extremes for large marine ecosystem regions of Northern Oceans. *Elementa.* **6**, 9 (2018).

23. D. J. Amaya, M. A. Alexander, A. Capotondi, C. Deser, K. B. Karnauskas, A. J. Miller, N. J. Mantua, Are long-term changes in mixed layer depth influencing North Pacific Marine heatwaves? *Bull. Am. Meteorol. Soc.* **102**, S59–S66 (2021).

24. A. Capotondi, M. A. Alexander, N. A. Bond, E. N. Curchitser, J. D. Scott, Enhanced upper ocean stratification with climate change in the CMIP3 models. *J. Geophys. Res. Ocean.* **117**, C04031 (2012).

25. H. J. Freeland, Evidence of change in the winter mixed layer in the Northeast Pacific Ocean: A problem revisited. *Atmos. - Ocean.* **51**, 126–133 (2013).

26. F. Liu, J. Lu, Y. Luo, Y. Huang, F. Song, On the oceanic origin for the enhanced seasonal cycle of SST in the midlatitudes under global warming. *J. Climate* **33**, 8401–8413 (2020).

27. G. Li, L. Cheng, J. Zhu, K. E. Trenberth, M. E. Mann, J. P. Abraham, Increasing ocean stratification over the past half-century. *Nat. Clim. Chang.* **10**, 1116–1123 (2020).

28. V. Eyring, S. Bony, G. A. Meehl, C. A. Senior, B. Stevens, R. J. Stouffer, K. E. Taylor, Overview of the coupled model intercomparison project phase 6 (CMIP6) experimental design and organization. *Geosci. Model Dev.* **9**, 1937–1958 (2016).

29. K. Hasselmann, Stochastic climate models Part I. Theory. *Tellus.* **28**, 473–485 (1976).

30. J. E. Kay, C. Deser, A. Phillips, A. Mai, C. Hannay, G. Strand, J. M. Arblaster, S. C. Bates, G. Danabasoglu, J. Edwards, M. Holland, P. Kushner, J. F. Lamarque, D. Lawrence, K. Lindsay, A. Middleton, E. Munoz, R. Neale, K. Oleson, L. Polvani, M. Vertenstein, The community earth system model (CESM) large ensemble project: A community resource for studying climate change in the presence of internal climate variability. *Bull. Am. Meteorol. Soc.* **96**, 1333–1349 (2015).

31. T. Bayr, C. Wengel, M. Latif, D. Dommenget, J. Lübbecke, W. Park, Error compensation of ENSO atmospheric feedbacks in climate models and its influence on simulated ENSO dynamics. *Climate Dynam.* **53**, 155–172 (2019).

32. J. Ma, S. P. Xie, Y. Kosaka, Mechanisms for tropical tropospheric circulation change in response to global warming. *J. Climate* **25**, 2979–2994 (2012).

33. T. Andrews, J. M. Gregory, M. J. Webb, The dependence of radiative forcing and feedback on evolving patterns of surface temperature change in climate models. *J. Clim.* **28**, 1630–1648 (2015).

34. J. Li, D. W. J. Thompson, Widespread changes in surface temperature persistence under climate change. *Nature* **599**, 425–430 (2021).

35. C. A. Stock, K. Pegion, G. A. Vecchi, M. A. Alexander, D. Tommasi, N. A. Bond, P. S. Fratantoni, R. G. Gudgel, T. Kristiansen, T. D. O'Brien, Y. Xue, X. Yang, Seasonal sea surface temperature anomaly prediction for coastal ecosystems. *Prog. Oceanogr.* **137**, 219–236 (2015).

36. M. G. Jacox, M. A. Alexander, S. Siedlecki, K. Chen, Y. O. Kwon, S. Brodie, I. Ortiz, D. Tommasi, M. J. Widlansky, D. Barrie, A. Capotondi, W. Cheng, E. Di Lorenzo, C. Edwards, J. Fiechter, P. Fratantoni, E. L. Hazen, A. J. Hermann, A. Kumar, A. J. Miller, D. Pirhalla, M. Pozo Buil, S. Ray, S. C. Sheridan, A. Subramanian, P. Thompson, L. Thorne, H. Annamalai, K. Aydin, S. J. Bograd, R. B. Griffis, K. Kearney, H. Kim, A. Mariotti, M. Merrifield, R. Rykaczewski, Seasonal-to-interannual prediction of North American coastal marine ecosystems: Forecast methods, mechanisms of predictability, and priority developments. *Prog. Oceanogr.* **183**, 102307 (2020).

37. N. A. Bond, M. F. Cronin, H. Freeland, N. Mantua, Causes and impacts of the 2014 warm anomaly in the NE Pacific. *Geophys. Res. Lett.* **42**, 3414–3420 (2015).

38. L. M. Cavole, A. M. Demko, R. E. Diner, A. Giddings, I. Koester, C. M. L. S. Pagniello, M. L. Paulsen, A. Ramirez-Valdez, S. M. Schwenck, N. K. Yen, M. E. Zill, P. J. S. Franks,

Biological impacts of the 2013–2015 warm-water anomaly in the northeast Pacific: Winners, losers, and the future. *Oceanography* **29**, 273–285 (2016).

39. M. G. Jacox, M. A. Alexander, N. J. Mantua, J. D. Scott, G. Hervieux, R. S. Webb, F. E. Werner, Forcing of multiyear extreme ocean temperatures that impacted California current living marine resources in 2016. *Bull. Am. Meteorol. Soc.* **99**, S27–S33 (2018).

40. J. F. Piatt, J. K. Parrish, H. M. Renner, S. K. Schoen, T. T. Jones, M. L. Arimitsu, K. J. Kuletz, B. Bodenstein, M. García-Reyes, R. S. Duerr, R. M. Corcoran, R. S. A. Kaler, G. J. McChesney, R. T. Golightly, H. A. Coletti, R. M. Suryan, H. K. Burgess, J. Lindsey, K. Lindquist, P. M. Warzybok, J. Jahncke, J. Roletto, W. J. Sydeman, Extreme mortality and reproductive failure of common murres resulting from the northeast Pacific marine heatwave of 2014-2016. *PLOS ONE* **15**, e0226087 (2020).

41. D. J. Amaya, A. J. Miller, S. P. Xie, Y. Kosaka, Physical drivers of the summer 2019 North Pacific marine heatwave. *Nat. Commun.* **11**, 1903 (2020).

42. D. A. Smale, T. Wernberg, E. C. J. Oliver, M. Thomsen, B. P. Harvey, S. C. Straub, M. T. Burrows, L. V. Alexander, J. A. Benthuysen, M. G. Donat, M. Feng, A. J. Hobday, N. J. Holbrook, S. E. Perkins-Kirkpatrick, H. A. Scannell, A. Sen Gupta, B. L. Payne, P. J. Moore, Marine heatwaves threaten global biodiversity and the provision of ecosystem services. *Nat. Clim. Chang.* **9**, 306–312 (2019).

43. M. G. Jacox, M. A. Alexander, S. J. Bograd, J. D. Scott, Thermal displacement by marine heatwaves. *Nature* **584**, 82–86 (2020).

44. G. Hervieux, M. A. Alexander, C. A. Stock, M. G. Jacox, K. Pegion, E. Becker, F. Castruccio, D. Tommasi, More reliable coastal SST forecasts from the North American multimodel ensemble. *Climate Dynam.* **53**, 7153–7168 (2019).

45. M. Hoerling, A. Kumar, The perfect ocean for drought. *Science (80-.).* **229**, 691–694 (2003).

46. F. J. Doblas-Reyes, J. García-Serrano, F. Lienert, A. P. Biescas, L. R. L. Rodrigues, Seasonal climate predictability and forecasting: Status and prospects. *Wiley Interdiscip. Rev. Clim. Chang.* **4**, 245–268 (2013).

47. S. D. Schubert, R. E. Stewart, H. Wang, M. Barlow, E. H. Berbery, W. Cai, M. P. Hoerling, K. K. Kanikicharla, R. D. Koster, B. Lyon, A. Mariotti, C. R. Mechoso, O. V. Müller, B. Rodriguez-Fonseca, R. Seager, S. I. Senevirante, L. Zhang, T. Zhou, Global meteorological drought: A synthesis of current understanding with a focus on sst drivers of precipitation deficits. *J. Climate* **29**, 3989–4019 (2016).

48. D. M. Smith, A. A. Scaife, B. P. Kirtman, What is the current state of scientific knowledge with regard to seasonal and decadal forecasting? *Environ. Res. Lett.* **7**, 015602 (2012).

49. B. Wang, J. Y. Lee, B. Xiang, Asian summer monsoon rainfall predictability: A predictable mode analysis. *Climate Dynam.* **44**, 61–74 (2014).

50. B. Wang, B. Xiang, J. Li, P. J. Webster, M. N. Rajeevan, J. Liu, K. J. Ha, Rethinking Indian monsoon rainfall prediction in the context of recent global warming. *Nat. Commun.* **6**, 7154 (2015).

51. S. Y. Yim, B. Wang, W. Xing, Peak-summer East Asian rainfall predictability and prediction part II: Extratropical East Asia. *Climate Dynam.* **47**, 15–30 (2016).

52. W. Xing, B. Wang, S. Y. Yim, K. J. Ha, Predictable patterns of the May-June rainfall anomaly over East Asia. *J. Geophys. Res.* **122**, 2203–2217 (2017).

53. J. Li, B. Wang, Predictability of summer extreme precipitation days over eastern China. *Climate Dynam.* **51**, 1–12 (2018).

54. X. Luo, B. Wang, How predictable is the winter extremely cold days over temperate East Asia? *Climate Dynam.* **48**, 2557–2568 (2017).

55. X. Luo, B. Wang, Predictability and prediction of the total number of winter extremely cold days over China. *Climate Dynam.* **50**, 1769–1784 (2018).

56. M. Gao, B. Wang, J. Yang, W. Dong, Are peak summer sultry heat wave days over the Yangtze-Huaihe River basin predictable? *J. Climate* **31**, 2185–2196 (2018).

57. I. M. Held, B. J. Soden, Robust responses of the hydrological cycle to global warming. *J. Climate* **19**, 5686–5699 (2006).

58. D. L. Swain, B. Langenbrunner, J. D. Neelin, A. Hall, Increasing precipitation volatility in twenty-first-century California. *Nat. Clim. Chang.* **8**, 427–433 (2018).

59. N. C. Stenseth, A. Mysterud, G. Ottersen, J. W. Hurrell, K.-S. Chan, M. Lima, Ecological effects of climate fluctuations. *Science (80-.).* **297**, 1292–1296 (2002).

60. P. van der Sleen, P. A. Zuidema, J. Morrongiello, J. L. J. Ong, R. R. Rykaczewski, W. J. Sydeman, E. Di Lorenzo, B. A. Black, Interannual temperature variability is a principal driver of low-frequency fluctuations in marine fish populations. *Commun. Biol.* **5**, 28 (2022).

61. A. E. Punt, T. A'mar, N. A. Bond, D. S. Butterworth, C. L. de Moor, J. A. A. De Oliveira, M. A. Haltuch, A. B. Hollowed, C. Szuwalski, Fisheries management under climate and environmental uncertainty: Control rules and performance simulation. *ICES J. Mar. Sci.* **71**, 2208–2220 (2014).

62. E. R. Pianka, On r-and K-selection. *Am. Nat.* **104**, 592–597 (1970).

63. D. Reznick, M. J. Bryant, F. Bashey, r- and K-selection revisited: The role of population regulation in life-history evolution. *Ecology* **83**, 1509–1520 (2002).

64. S. Hočevar, J. A. Hutchings, A. Kuparinen, Multiple-batch spawning as a bet-hedging strategy in highly stochastic environments: An exploratory analysis of Atlantic cod. *Evol. Appl.* **14**, 1980–1992 (2021).

65. B. C. O'Neill, C. Tebaldi, D. P. Van Vuuren, V. Eyring, P. Friedlingstein, G. Hurtt, R. Knutti, E. Kriegler, J. F. Lamarque, J. Lowe, G. A. Meehl, R. Moss, K. Riahi, B. M.

Sanderson, The Scenario Model Intercomparison Project (ScenarioMIP) for CMIP6. *Geosci. Model Dev.*. **9**, 3461–3482 (2016).

66. J. F. Lamarque, P. P. Kyle, M. Meinshausen, K. Riahi, S. J. Smith, D. P. van Vuuren, A. J. Conley, F. Vitt, Global and regional evolution of short-lived radiatively-active gases and aerosols in the representative concentration pathways. *Clim. Change* **109**, 191–212 (2011).

67. N. A. Rayner, Global analyses of sea surface temperature, sea ice, and night marine air temperature since the late nineteenth century. *J. Geophys. Res.* **108**, 4407 (2003).

68. R. W. Reynolds, T. M. Smith, C. Liu, D. B. Chelton, K. S. Casey, M. G. Schlax, Daily high-resolution-blended analyses for sea surface temperature. *J. Climate* **20**, 5473–5496 (2007).

69. V. Banzon, T. M. Smith, T. Mike Chin, C. Liu, W. Hankins, A long-term record of blended satellite and in situ sea-surface temperature for climate monitoring, modeling and environmental studies. *Earth Syst. Sci. Data* **8**, 165–176 (2016).

70. E. Hawkins, R. Sutton, The potential to narrow uncertainty in regional climate predictions. *Bull. Am. Meteorol. Soc.* **90**, 1095–1108 (2009).

71. F. Lehner, C. Deser, N. Maher, J. Marotzke, E. M. Fischer, L. Brunner, R. Knutti, E. Hawkins, Partitioning climate projection uncertainty with multiple large ensembles and CMIP5/6. *Earth Syst. Dynam.* **11**, 491–508 (2020).

72. A. Storto, S. Masina, C-GLORSv5: An improved multipurpose global ocean eddy-permitting physical reanalysis. *Earth Syst. Sci. Data* **8**, 679–696 (2016).
